# Prognostic Factors Predict Oncological Outcome in Older Patients With Head and Neck Cancer Undergoing Chemoradiation Treatment

**DOI:** 10.3389/fonc.2020.566318

**Published:** 2021-02-23

**Authors:** Carmen Stromberger, Berna Yedikat, Annekatrin Coordes, Ingeborg Tinhofer, Goda Kalinauskaite, Volker Budach, Sebastian Zschaeck, Jan-Dirk Raguse, Grzegorz Kofla, Max Heiland, Aksana Stsefanenka, Benedicta Beck-Broichsitter, Steffen Dommerich, Carolin Senger, Marcus Beck

**Affiliations:** ^1^Department of Radiation Oncology, Charité–Universitätsmedizin Berlin, corporate member of Freie Universität Berlin, Humboldt-Universität zu Berlin, Berlin, Germany; ^2^Berlin Institute of Health (BIH), Berlin, Germany; ^3^Department of Otorhinolaryngology, Head and Neck Surgery, Charité–Universitätsmedizin Berlin, corporate member of Freie Universität Berlin, Humboldt-Universität zu Berlin, Berlin, Germany; ^4^German Cancer Research Center (DKFZ), Heidelberg, Germany; ^5^German Cancer Consortium (DKTK) partner site Berlin, Berlin, Germany; ^6^Department of Oral and Maxillofacial Surgery, Charité–Universitätsmedizin Berlin, corporate member of Freie Universität Berlin, Humboldt-Universität zu Berlin, Berlin, Germany; ^7^Department of Oncology, Charité–Universitätsmedizin Berlin, corporate member of Freie Universität Berlin, Humboldt-Universität zu Berlin, Berlin, Germany

**Keywords:** head and neck cancer (HNC), head and neck squamous cell carcinoma (HNSCC), older patients, head and neck cancer, survival, chemoradiation, volumetric modulated arc therapy (VMAT), radiation

## Abstract

**Purpose:**

Older patients with head and neck cancer (HNC) represent a challenging group, as frailty and comorbidities need to be considered. This study aimed to evaluate the efficacy and side effects of curative and palliative (chemo) radiation ([C]RT) with regard to basic geriatric screening in older patients.

**Methods:**

This study included HNC patients aged ≥70 years who were treated with curative or palliative (C)RT. Clinicopathological data including Charlson Comorbidity Index (CCI), Karnofsky performance status (KPS), and treatment data were analyzed as predictors of overall survival (OS).

**Results:**

A total of 271 patients (median age, 74 years) were enrolled. The majority had UICC stage III/IV (90%) and underwent curative treatment (85.2%). A total of 144 (53.1%) patients received definitive and 87 (32.1%) had adjuvant (C)RT. Overall, 40 patients (14.8%) received palliative (C)RT. Median follow-up duration (curative setting) was 87 months, and the 2- and 5-year OS rates were 57.8 and 35.9%, respectively. Median OS was significantly different for age ≤75 *vs.* >75 years, CCI <6 *vs.* ≥6, KPS ≥70 *vs.* <70%, Tx/T1/T2 v*s*. T3/T4, and adjuvant *vs.* definitive (C)RT, respectively. Age 70–75 years (p = 0.004), fewer comorbidities when CCI < 6 (p = 0.014), good KPS ≥ 70% (p = 0.001), and adjuvant (C)RT (p = 0.008) independently predicted longer survival. Palliative RT resulted in a median OS of 4 months.

**Conclusion:**

Older age, lower KPS, higher CCI, and definitive (C)RT are indicators of worse survival in older patients with HNC treated curatively. Without a comprehensive geriatric assessment in patients aged >75 years, the KPS and CCI can be useful tools to account for “fitness, vulnerability or frailty” to help in treatment decision-making.

## Introduction

In developed countries, older generations are becoming the dominant demographic groups, and the number of oncological patients is increasing. In addition, the number of deaths among cancer patients aged 80 years and older is almost twice as often as from heart disease than from cancer (1). How to deal with older oncological patients? The question being frequently asked by oncologists, surgeons, and radiation oncologists in recent years. Worldwide, head and neck cancer (HNC) cases account for approximately 650,000 cases annually (1). In Europe, it accounts for an estimated 4% of all cancer incidences (2). Curative treatment of locally advanced (LA) HNC with surgery and/or chemoradiation (CRT) is commonly associated with severe treatment-related toxicity, and recent findings suggest that older patients >70 years with locally advanced head and neck squamous cell carcinoma (HNSCC) have an increased 90-day mortality after concurrent CRT (3). Furthermore, older patients with HNC have a 1-year mortality rate of 42.3%, and malnutrition and immobility seem to be independent negative predictors for worse survival (4). In addition, HNC patients aged ≥70 years are under-represented in prospective randomized trials. With limited data on older patients, as only approximately 7.5% of patients were aged 71 years or older, a meta-analysis showed that the estimated survival benefit of approximately 6.5% at 5 years with simultaneous platinum-based CRT for LA HNC patients significantly disappeared with increasing age (p = 0.003) (5). Some recommendations for screening tools in the geriatric assessment of older patients with cancer are available (6, 7). However, in daily oncological practice in many countries, a comprehensive geriatric assessment (CGA) is seldom part of the decision-making process in a multidisciplinary approach for older patients with HNC. The selection of older patients fit for an intensive multimodal treatment or moderately suitable for less intensive curative radiotherapy alone (RT), and frail older patients appropriate for palliative RT or best supportive care needs careful evaluation to achieve the greatest benefit for the individual patient.

The aim of this study was to evaluate the “treatment decision” made by an interdisciplinary team in terms of outcome and predictive factors for survival in older patients with HNC treated with curative or palliative (chemo)radiation (C)RT based on comorbidities and performance status.

## Materials and Methods

### Study Design and Setting

After receiving institutional review board approval (EA2/140/19), data from all older patients (age ≥ 70 years) with histologically confirmed head and neck malignancies (oro-/hypo-/nasopharynx, oral cavity, larynx nasal/paranasal sinuses, salivary glands, cancer of unknown primary (CUP) treated at the Department of Radiation Oncology, Charité–Universitätsmedizin Berlin, between January 2005 and October 2015 were reviewed. Tumor stage, nodal stage, UICC stage (7^th^ edition TNM classification), Karnofsky performance status (KPS), Charlson Comorbidity Index (CCI), curative definitive, and adjuvant (C)RT with resection margin (R0, R1 or close <5 mm) and/or extranodal extension (ENE), palliative RT, RT technique, and total RT dose were analyzed retrospectively from electronic and paper charts. Comorbidity conditions were evaluated with the use of the CCI, the sum of a point-based scoring system based on 19 pathologic conditions (8). A web-based tool for the prediction of age-adjusted 10-year survival in patients with multiple comorbidities was used to calculate CCI (9). Acute treatment-related toxicity was scored according to Common Terminology Criteria for Adverse Events (CTCAE) Version 4.0 (10).

### Follow-Up and Survival

All patients had follow-up examinations scheduled at three months post treatment and 6-monthly thereafter for the first five years. Physical examination and yearly computed tomography (CT) or magnetic resonance imaging of the head and neck region, thorax, and abdomen were part of the follow-up. Overall survival (OS) was calculated from the beginning of the (C)RT until the last follow-up or death from any cause. In addition, survival data were collected from the tumor registry (Gießener Tumordokumentationssystem, GTDS) and the Charité Comprehensive Cancer Center.

### Statistical Analysis

Descriptive statistics of patient baseline and tumor characteristics between curative adjuvant versus definitive (C)RT were performed using the chi-square test to account for statistical differences. As primary outcome parameter, OS was analyzed according to intention to treat for patients with curative definitive or adjuvant (C)RT and with palliative (C)RT. OS rates were analyzed by the Kaplan–Meier method, and the log-rank test was used to assess statistical significance. For univariate and multivariate analyses, Cox proportional hazards models were used. Cut-off values for age (area under the curve [AUC] 0.608, p = 0.005; 95% confidence interval [CI] 0.535–0.681) and CCI (AUC 0.631, p = 0.001, 95% CI 0.558–0.703) were confirmed by receiver operating characteristic (ROC) analysis at four years for death. The following clinicopathological and treatment parameters were recorded and analyzed: sex (male *vs.* female), age (≤75 *vs.* >75 years), CCI (<6 *vs.* ≥6), KPS (≥70 *vs.* <70%), T-classification (Tx/T1/T2 *vs.* T3/T4), N-classification (negative *vs.* positive), UICC stage (I/II *vs.* III/IV), tumor site (oropharynx *vs.* oral cavity *vs.* larynx *vs.* hypopharynx *vs.* salivary glands *vs.* nasal/paranasal sinuses *vs.* nasopharynx *vs.* CUP), histology, curative (C)RT (adjuvant *vs.* definitive), and chemotherapy (no *vs.* yes). All survival-associated variables (p < 0.1) in the univariate analysis were further investigated using the Cox multivariate regression model with stepwise backward logistic regression. P values <0.05 were considered statistically significant. Statistical analyses were performed using IBM SPSS Statistics, Version 25.2 (IBM Corp., Armonk, NY, USA).

## Results

Overall, 271 HNC patients aged ≥70 years fulfilled the criteria: 194 were male (71.6%) and 77 were female (28.4%). The median age at diagnosis was 74 years (range, 70–92 years), and 111 patients (41.0%) were 76 years or older. Good KPS (100–70%) was present in 178 patients (65.7%). CCI <6 was observed in 139 (51.3%) patients, and CCI ≥6 in 132 patients (48.7%). Tumor stage T3/T4 and/or nodal positive stage was reported in 193 patients (71.2%). Distant metastases were observed in 15 patients (5.5%). Advanced UICC stage III/IV was present in 245 patients (90.4%). The majority of patients (95.6%) had squamous cell carcinoma (SCC) (see **Table 1**).

**Table 1:** Characteristics of older patients with head and neck cancer (n = 271).

**Variable**		**n = 271 (%)**
**Mean age at diagnosis of HNC, years (SD, range)**		75.55 (5.113, 70–92)
**Age, years**	70–75	162 (59.8)
	76–80	63 (23.2)
	81–85	32 (11.8)
	>85	14 (5.2)
**Sex**	male	194 (71.6)
**Charlson Comorbitidy Index (CCI)**	Mean, score (SD, range)	5.76 (2.146, 3–14)
	3	36 (13.3)
	4	60 (22.1)
	5	43 (15.9)
	6	44 (16.2)
	7	32 (11.8)
	8	21 (7.7)
	9	17 (6.3)
	10	13 (4.8)
	11	3 (1.1)
	12	1 (0.4)
	14	1 (0.4)
**Karnofsky Performance status (KPS)**	100%	5 (1.8)
	90%	28 (34.3)
	80%	50 (18.5)
	70%	95 (35.1)
	60%	68 (25.1)
	50%	19 (7)
	40%	5 (1.8)
	30%	1 (0.4)
***HNC characteristics***		
**Tumor site**	Oropharynx	98 (36.2)
	Oral cavity	73 (26.9)
	Larynx	39 (14.4)
	Hypopharynx	29 (10.7)
	Salivary glands	14 (5.2)
	Nasal/paranasal sinus	11 (4.1)
	Nasopharynx	4 (1.5)
	CUP	3 (1.1)
**T-classification**	T1	21 (7.7)
	T2	52 (19.2)
	T3	84 (31.0)
	T4	109 (40.2)
	Tx	6 (2.2)
	unknown	3 (1.1)
**N-classification**	N0	71 (26.2)
	N1	38 (14.0)
	N2	144 (53.1)
	N2a	10 (3.7)
	N2b	76 (28.0)
	N2c	55 (20.3)
	N2	4 (1.5)
	N3	7 (2.6)
	Nx	7 (2.6)
	N+ (not specified)	3 (1.1)
**M-classification**	Positive	15 (5.5)
**UICC stage 7^th^**	I	4 (1.5)
	II	21 (7.7)
	III	53 (19.6)
	IV	192 (70.8)
	unknown	1 (0.4)
**Histology**	SCC	259 (95.6)
	Adeno cacinoma	5 (1.8)
	Adenoid cystic carcinoma	3 (1.1)
	other	4 (1.5)
**Resection margin**	R0	41 (47.1)
	R1, or close <5mm	18 (20.7)
	ENE	17 (19.5)
	R1 & ENE	6 (6.9)
	missing	5 (5.7)
**Therapy**		
**Radiotherapy**	Definitive	144 (53.1)
	completed	131 (91.0)
	Mean dose, Gy (SD, range)	66.37 (12.957, 0-74.4)
	Median dose, Gy	70.4
	Adjuvant	87 (32.1)
	completed	81 (93.1)
	Mean dose, Gy (SD, range)	59.20 (9.928, 6-70.5)
	Median dose, Gy	63.70
	Palliative	40 (14.8)
	completed	31 (77.4)
	Mean dose, Gy (SD, range)	34.62 (13.135, 9-50)
	Median dose, Gy	42.00
**Systemic therapy**		159 (58.7)
	Cisplatin ± 5-FU	71 (44.7)
	Mitomycin C ± 5-FU	66 (41.5)
	Cetuximab	16 (10.1)
	TPF	3 (1.9)
	Carboplatin	2 (1.3)
	Drug unknown	1 (0.6)
**Radiotherapy technique**	VMAT/IMRT	247 (91.1)
	SIB	82 (30.3)
	3D-CRT/2D-RT	24 (8.9)

HNC, head and neck cancer; SD, standard deviation; CUP, cancer of unknown primary; UICC, Union for International Cancer Control; SCC, squamous cell carcinoma; R, resection margin; ENE, extracapsular extension; 5-FU, 5-flurouracil; TPF, taxan, platinum, 5-flurouracil; VMRT, volumetric modulated arc therapy; IMRT, intensity modulated radiotherapy; SIB, simultaneous integrated boost; 3D-CRT, 3 conformal radiotherapy; 2D-RT 2 two-dimensional radiotherapy.

In the curative setting (n = 231), 62 patients (43.1%) with definitive and 21 (24.1%) with adjuvant (C)RT were older than 75 years, with the median age being 74 years (range, 70–92) *versus* 73 years (range, 70–84 years, p = 0.398). Age categories (years, definitive *vs.* adjuvant [C] RT) 70–75: 82 (56.9%) *vs.* 66 (75.9%), 76–80: 41 (28.5%) *vs.* 13 (14.9%), 81–85: 14 (9.7%) *vs.* 8 (9.2%), >85: 7 (4.9%) *vs.* 0 (0%) were significantly different (p = 0.009). Baseline characteristics for KPS, CCI, tumor site, N-classification, and UICC stage were not significantly different in HNC patients with definitive and adjuvant treatment intent. In contrast, advanced T3/4-classification was significantly more frequent for definitive treatment (115 patients, 79.9%) than for adjuvant treatment (43 patients, 49.4%; p < 0.001). Oropharyngeal cancer (OPC) was present in 35 (42.5%) patients receiving adjuvant (C)RT and 52 (36.1%) receiving definitive (C)RT. Although data on human papilloma virus (HPV) status and smoking were not available for our patients, a previous systematic survey on the trends in HPV prevalence and smoking behavior at the Charité by our group revealed persistently high rates of smokers in the OPC group and a slow but continuous increase in the prevalence of HPV-driven low-risk OPC (2004: 6%, 2013: 23%) (11). Curative treatment was indicated in 231 patients (85.2%) and completed in 212 patients (91.8%). Only 13 patients (9.0%) receiving definitive RT and six patients (6.9%) receiving adjuvant RT did not finish treatment as planned. In the curative setting, concurrent systemic therapy was administered to 147 patients (63.6%), 110 patients (74.8%) had definitive CRT, and 37 patients (25.2%) received adjuvant CRT. Forty-one (47.1%) of the adjuvant therapy patients had postoperative high-risk features, and in 90.2% of these cases, concurrent CRT was scheduled. Cisplatin or mitomycin C ± 5-fluorouracil was used in 51 (46.4%) and 45 patients (40.9%) in the definitive treatment, respectively. Adjuvant CRT with concurrent cisplatin was administered to 20 patients (54.1%). Other drugs were administered to 31 (21.1%) of the curative patients (**Table 1**).

In the palliative treatment intent group (n = 40), 25 patients (62.5%) were 76 years or older, 29 (72.5%) had a CCI ≥6, and 32 patients (80%) had a KPS <70%. Thirty-six patients (90%) had a UICC Stage IV, and 11 patients (27.5%) suffered from distant metastasis from HNC. Nine patients (22.5%) received a total RT dose of less than 20 Gy. Palliative concurrent CRT was administered to 12 patients (30%). Mitomycin C was administered to 11 patients (91.7%) and cetuximab to one patient (8.3%).

In general, the majority of all patients (91.1%) had modern RT treatment. A total of 175 patients underwent volumetric modulated arc therapy (VMAT, 64.6%) and 72 patients received intensity-modulated radiotherapy (IMRT, 26.6%), and 30.3% of patients were treated with a simultaneous integrated boost (SIB).

Detailed patient, tumor, and treatment characteristics are shown in **Table 1**.

### Treatment Outcome

#### Curative Treatment

**Figure 1:**
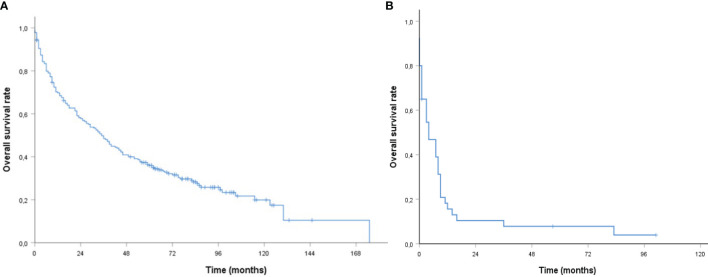
**|** Kaplan–Meier curves of older patients with head and neck cancer **(A)** curative **(B)** palliative (chemo) radiation with respect to overall survival.

The median follow-up for survivors was 87 months (95% CI, 79.09–94.91, range 0–175) with 2- and 5-year OS rates of 57.8 and 35.9%, respectively. The median OS was 35 months (95% CI, 27.36–42.65) (see **Figure 1A**). The median OS was 46 *vs.* 23 months for age ≤75 *vs.* >75 years (95% CI, 29.97–62.03 *vs.* 8.93–37.08, p < 0.001), 44 *vs.* 27 months for a CCI <6 *vs.* ≥6 (95% CI, 27.43–60.57 *vs.* 15.52–38.48, p= 0.001), 44 *vs.* 10 months with a KPS ≥70 *vs.* <70% (95% CI, 33.66–54.34 *vs.* 3.39–16.61, p < 0.001), 52 *vs.* 27 months with Tx/T1/T2 *vs.* T3/T4 classification (95% CI, 36.38–67.62 *vs.* 20.00–34.00, p = 0.036), and 52 *vs.* 23 months with adjuvant *vs.* definitive (C)RT (95% CI, 34.05–69.96 *vs.* 14.86–1.14, p = 0.003).

No significant differences were observed in terms of median OS for sex (34 *vs.* 40 months, male *vs.* female, 95% CI, 25.13–42.87 *vs.* 16.67–3.33, p = 0.2), N-negative *vs.* N-positive (36 *vs.* 34 months, 95% CI, 16.54–55.46 *vs.* 25.23–42.77, p = 0.921), and UICC stage I/II *vs.* III/IV (36 *vs.* 34 months, 95% CI, 29.84–42.16 *vs.* 25.17–42.83, p = 0.971), and curative CRT *vs.* RT (42 *vs.* 28 months, 95% CI, 32.41–51.59 *vs.* 16.25–39.75, p = 0.528). Moreover, the median OS was not different with definitive CRT *vs.* definitive RT alone, but showed a trend towards improved OS by CRT (25 compared to 13 months, 95% CI, 13.32–36.68 *vs.* 0–30.92, p = 0.085).

**Figure 2:**
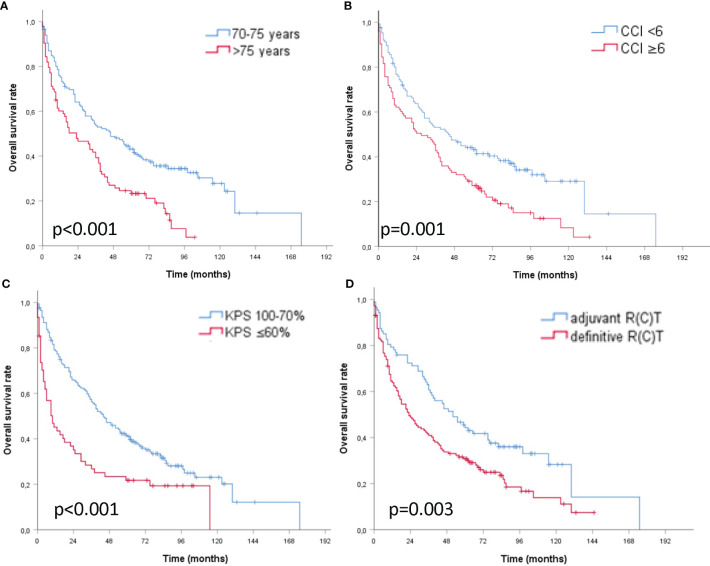
**|** Kaplan–Meier curves of older patients with head and neck cancer and curative (chemo) radiation **(A)** age ≤75 *vs.* >75 years **(B)** CCI <6 *vs.* ≥6 points **(C)** KPS ≥70 *vs.* <70% **(D)** adjuvant *vs.* definitive (chemo) radiation with respect to overall survival.

The 2-year OS rates were 64.1 *vs.* 46.6% for patients aged ≤75 *vs.* >75 years, 63.8 *vs.* 50.8% with a CCI <6 *vs.* ≥6, 65.9 *vs.* 35.1% with a KPS ≥70 *vs.* <70%, and 72.4 *vs.* 48.8% with adjuvant *vs.* definitive RT, respectively. The 5-year OS rates were 43.1 *vs.* 23.3% aged ≤75 *vs.* >75 years, 43.2 *vs.* 27.1% with a CCI <6 *vs.* ≥6, 41.0 *vs.* 21.7% with a KPS ≥70 *vs.* <70%, and 44.3 *vs.* 30.8% adjuvant *vs.* definitive (C)RT, respectively (**Figures 2A–D**). Results of the univariate and multivariate Cox regression analyses are shown in **Table 2**.

**Table 2:** **|** Univariate and multivariate analysis of clinicopathological and treatment parameters associated with overall survival (OS) in older patients with HNC and curative intended (chemo) radiation.

Univariate Analysis					
Variable		N = 231	HR	95% CI	p-value
Sex	Male	168	0.799	0.565–1.131	0.204
	Female	63			
Age (years)	≤75	148	1.822	1.332–2.493	0.000
	>75	83			
CCI	<6	128	1.662	1.225–2.254	0.001
	≥6	103			
KPS	≥70%	170	1.921	1.373–2.687	0.000
	<70%	61			
T-classification	Tx/T1/T2	73	1.422	1.018–1.986	0.035
	T3/T4	158			
N-classification	negative	67	0.984	0.706–1.370	0.922
	positive	164			
UICC stage 7^th^	I/II	24	0.991	0.607–1.618	0.971
	III/IV	205			
Tumor site	Oropharynx	87	1.032	0.956–1.113	0.421
	Oral cavity	57			
	Larynx	36			
	Hypopharynx	27			
	Salivary glands	10			
	Nasal/paranasal sinus	7			
	Nasopharynx	4			
	CUP	3			
RT treatment	adjuvant	87	1.614	1.170–2.225	0.004
	definitive	144			
Chemotherapy	No	84	1.106	0.806–1.519	0.532
	Yes	147			
**Multivariate Analysis**					
**Variable**	** **	**N = 231**	**HR**	**95% CI**	**p-value**
Age (years)	≤75	148	1.606	1.167–2.212	0.004
	>75	83			
CCI	<6	128	1.470	1.080–2.001	0.014
	≥6	103			
KPS	≥70%	170	1.896	1.342–2.679	0.001
	<70%	61			
T-classification	Tx/T1/T2	74	1.133	0.792–1.620	0.492
	T3/T4	157			
RT treatment	adjuvant	87	1.560	1.118–2.176	0.008
	definitive	144			

HNC, head and neck cancer; HR, hazard ratio, 95% CI, 95% confidence interval; CCI, Charlson Comorbidity Index; KPS, Karnofsky performance status; CUP, cancer of unknown primary; RT, radiotherapy.

#### Palliative Treatment

In patients with palliative treatment intent (n = 40), the estimated median OS was four months (95% CI, 0.39–7.61, **Figure 1B**). OS rates were not different for age groups [hazard ratio (HR) 1.30, 95% CI, 0.65–2.61, p = 0.460], CCI (HR 1.37, 95% CI, 0.66–.84, p = 0.400), KPS (HR 1.65, 95% CI, 0.75–2.66, p = 0.212), or application of chemotherapy (HR 0.62, 95% CI, 0.31–1.27, p = 0.189).

### Treatment-Related Toxicity

#### Curative Treatment

Severe acute toxicity was observed as follows: mucositis grade (G) 3 in 27 (11.7%) and G4 in two (0.9%), dermatitis G3 in 23 (10%), dysphagia G3 and G4 in 87 (37.7%), and two (0.9%), dry mouth G3 in two (0.9%), fatigue G3 in one (0.4%), nausea G3 in one (0.4%), and pain G3 in 12 (5.2%) patients. Sepsis, pneumonia, and peritonitis were reported in five (2.2%) and 12 (5.2%) and three (1.3%) patients, respectively. All of these patients had definitive R(C)T. Two patients (0.9%) had a thrombosis, and one (0.4%) had an embolic lung event. Six patients (2.6%) died during the first 30 days of treatment (days 4–30), one from pneumonia, one from peritonitis, one from embolic lung event and bleeding, three (1.2%) deaths were due to tumor (progressive disease) and not treatment-related. In summary, three (1.2%) elderly patients died during the first 30 days of treatment.

#### Palliative Treatment

Acute toxicity G3 with mucositis was observed in three patients (7.5%), dysphagia in 16 (40%), dry mouth (2.5%), nausea (2.5%), and pain (12.5%). Two patients had sepsis (5%), one (2.5%) thrombosis, six patients (15%) had pneumonia, and one patient (2.5%) had peritonitis. Six patients (15%) died within 20 days of treatment (range, 6–17 days), one (2.5%) from sepsis, one (2.5%) from peritonitis, and four patients (10%) due to progressive disease and/or progressive weakness and deterioration.

## Discussion

This study assessed the OS of older patients with HNC treated with curative and palliative (C)RT with respect to comorbidities and performance scores in baseline assessments. Age has been investigated in previous studies of cancer (12, 13). In HNC patients with a mean age of 60 years, age was found to be a significant risk factor for non-cancer-related death, with an estimated HR of 1.05 per year (14). In a Japanese study, the risk of non-cancer-related death increased with an HR of 2.59 in patients aged 76 years and older, without a significant increase in HNC-related deaths (13). Kwon et al. reported a rate of 9.2% non-cancer-related death in predominantly (61.8%) surgically treated HNC patients, with respiratory events being the leading cause of death (12).

Our treatment recommendation elaborated in an interdisciplinary tumor board for each older patient was based on national and international treatment guidelines. Considering competing risks as a cause of death, older age (>75 years) before curative (C)RT was found to be a significant risk factor for survival in the univariate and multivariate analyses (HR 1.82, p > 0.001; HR 1.61, p = 0.004). Patients aged 70–75 years compared to 76 years or older had a gain of 23 months in median OS, and superior 2-year survival rates of 64.1 *versus* 46.6%. A recent retrospective German study on HNC patients with a median age of 72 years found a median OS of 40 *vs.* 22 months (*p* < 0.05) for patients aged 65–74 years and ≥ 75 years, but the age factor was not significant in the multivariate analysis (15).

To date, older patients aged ≥70 years with LA HNC have not been the primary candidates for treatment intensification in prospective clinical trials aiming at survival improvement because an increase in toxicities is a major concern with more aggressive treatment schedules (16). Moreover, treatment de-intensification with less toxic strategies while maintaining good oncological results has been the approach in recent years, *e.g.*, in HPV-positive oropharyngeal cancer patients of all ages and in dedicated studies for older patients with HNC (17–20). A meta-analysis showed that the survival benefit of standard concurrent chemoradiation in HNC patients aged 71 years or older significantly decreases with age, and the effect of poly-chemotherapy was not significantly different from that of mono-chemotherapy with cisplatin (5). We focused on HNC patients aged 70 years and older, with LA UICC stage and observed, in line with the data of Pignon et al. (5), that the addition of systemic therapy to curative definitive RT at this age had no additional effect on survival. However, our data need to be interpreted with caution since only 24% underwent radiotherapy alone. Up to 44% of patients had concurrent polychemotherapy with a potential effect on toxicity. We observed higher rates of severe acute toxicity such as dysphagia (44.4 *vs.* 28.7%) and dermatitis (12.5 *vs.* 5.8%) in the definitive (C)RT group, where 76% had simultaneous chemotherapy compared to patients in the adjuvant setting, with only approximately 43% receiving concurrent CRT.

A review on the management of older patients with locally advanced HNC suggests that, in addition to chronological age, the biological phenotype should be considered as a guide for the best treatment approach in terms of aggressiveness and likelihood of tolerance in terms of oncologic outcome and limitation of side effects (21). Frailty is defined as a reduced resistance or reserve to stressors, which leads to an increased risk of falls, disability, hospitalization, and death (22). Frail older patients with HNC should be treated aiming at symptom control through palliative RT or palliative care (21). As the majority of patients were aged 76 years or older, 80% had a reduced KPS and multiple comorbidities (CCI >6) in 72.5%, we observed a limited survival time of an estimated median 4 months after palliative RT with a mean dose of 34.6 Gy. Unfortunately, nine patients (22.5%) developed severe infections and six patients (15%) died within the first 17 days of palliative RT, while in the curative group, only 1.2% of older patients died within the first 30 days. Moreover, almost half of our palliative patients (40%) still had severe swallowing problems. This suggests that the selection of patients for palliative radiotherapy is crucial, and best supportive care needs to be considered a valid option for palliation. Lin et al. reported a 6.66% rate of 90-day mortality for patients with HNC at any age after the completion of definitive concurrent CRT. In addition to other factors, patients aged >70 years had a significantly increased risk of death (HR 2.18; 95% CI, 1.80-2.65; p  <  0.001) within 90 days after treatment (3). This 90-day mortality scoring system might be suitable for finding and selecting older patients who would benefit from a more conservative treatment than concurrent CRT (3). However, identifying the fit, moderate fit, and frail older patients remains challenging. By applying CGA in cancer patients aged 70 years or older, a Danish study group found that up to 87% of patients with cancer types often seen in older patients and often connected to comorbidity were either vulnerable or frail. Patients with lung cancer, colorectal cancer, or gastrointestinal cancer and 14.6% of HNC patients were included in this study (23). The utility of CGA for older patients with HNC is currently under evaluation in ongoing trials such as GEROP (NCT03053310) or ELAN-ONCOVAL (NCT03614936). Recently, an estimated mean time frame of 51 minutes per patient with an additional 5–10 min for calculating the scores has been described for a CGA (23). CGA was not performed in our study. As a baseline evaluation, we assessed the pre-radiotherapeutic patients’ performance status and comorbidities to estimate fitness, vulnerability, or frailty of the patient, and integrated these factors into the decision-making. For cancer outcome at any patient age, the performance status has been shown to be of prognostic significance (15, 24). Consistent with the literature, we found in our curative cohort that a reduced KPS of 60% or less resulted in decreased survival. A low KPS was particularly often present in our palliative cohort.

Beyond age and KPS, the presence of multiple comorbidities, in our study reflected by the CCI ≥6, showed a significant negative prognostic influence on OS with curative (C)RT in univariate and multivariate analyses. In a large Surveillance, Epidemiology, and End Results (SEER)-Medicare linked database study on older patients with HNC, a high prevalence of comorbidities such as hypertension, hyperlipidemia, chronic obstructive pulmonary disease, and diabetes at the time of diagnosis, and an increased likelihood of developing cancer-related comorbidities such as dysphagia, weight loss, or pneumonia after treatment was observed (25). Both findings are associated with a significantly increased risk of death in HNC patients at any age (26). Ryu and colleagues showed that comorbidities may compete for non-cancer risk factors in patients with HNSCC (27). Furthermore, comorbidities (14%), treatment-related acute (9%), and late toxicity (3%) accounted for 5-year non-cancer-related death rates of 4.1, 4.6, and 1.3%, respectively, in patients with LA HNC treated with CRT (14). Although acute toxicity was already high in our study, with the most common treatment-related side effects ≥ G3 being dysphagia in approximately 39%, 13% mucositis, and 11% dermatitis in curative patients, an underestimation of side effects needs to be assumed due to the retrospective nature of this study.

In our study, the curative treatment of choice was mostly definitive (C)RT, with 105 patients in UICC IV stage (72.9%), and the minority had surgery with adjuvant (C)RT. The adjuvant treated patients were significantly less likely to have an advanced T3/T4 stage and only 24.1% of the patients were older than 75 years. Furthermore, only approximately one-third (33%) of the adjuvant compared to two-thirds (67%) of the definitive treated patients presented with a CCI ≥6. All these factors might have favored the decision for surgery in older patients with less comorbidities. Perhaps, multimodal treatment with surgery independently predicted better survival than definitive RT. For older patients with oropharyngeal cancer, a SEER-Medicare data analysis found no difference in survival between surgery and definitive RCT (28). Recently, Yoshida and colleagues published data from the National Cancer Data Base on a large cohort of curative resected HNC patients aged 70 years or older with high-risk features (ENE and/or positive margin) treated with postoperative RT or CRT. Patients older than 74 years were significantly less frequently treated with CRT. They found 3-year OS rates of 52.4% in favor of concurrent CRT (p = 0.012) and an approximately 25% decreased risk of death through CRT vs. RT, especially with N2-N3 nodal involvement but not in nodal negative disease. Performance status, comorbidities, or side-effects were not investigated in the study (29).

Multiple limitations of our study need to be mentioned. In particular, the retrospective nature led to differences in the baseline characteristics of the patients in the definitive and postoperative (C)RT cohorts, which might have introduced a selection bias suggesting improved outcome with adjuvant treatment. Characteristics such as smoking history and the HPV, nutritional, or functional status were not available. Nevertheless, data from a previous study of the group on the prevalence of HPV and smoking habits suggest a relatively small proportion of HPV-associated low-risk OPC cases within our cohort (11). Furthermore, in the palliative setting, the relatively small number of patients also limited the validity of the analysis. A CGA or even an additional quick further performance measurement, such as using the timed “up and go” test (stand up from sitting, walk 3 m and return in less than 12 s), a useful tool that could have been easily integrated in the daily routine, was not performed (30). An analysis of loco-regional control, distant metastasis, or second cancer was not performed. We would like to emphasize the urgent need for prospective studies including geriatric assessment.

In summary, treatment for older patients with locally advanced HNC cancer remains a challenge, and multiple factors may influence survival. Older patients with HNC may benefit from curative more or less intense (C)RT with good compliance and decent survival; however, caution is advised in patients with advanced old age, reduced KPS, and multiple comorbidities. Frailty patients treated with radiotherapy have limited survival and symptom control. Prospective studies are awaited, but simple assessments could by now help to guide clinical decision-making.

## Data Availability Statement

The raw data supporting the conclusions of this article will be made available by the authors, without undue reservation.

## Ethics Statement

The studies involving human participants were reviewed and approved by the Institute Ethics Committee of the Charité–Universitätsmedizin Berlin, Germany (EA2/140/19). Written informed consent for participation was not required for this study in accordance with the national legislation and the institutional requirements.

## Author Contributions

CSt and MB provided ideas for the study. CSt and BY performed the analysis, and CSt drafted the manuscript. CSt calculated the underlying statistics. CSt, BY, AC, IT, GKa, VB, J-DR, ZS, GK, MH, AS, BB, SD, CSe, and MB were responsible for the treatment, collection of patient data, and follow-up. All authors contributed to the article and approved the submitted version.

## Conflict of Interest

The authors declare that the research was conducted in the absence of any commercial or financial relationships that could be construed as a potential conflict of interest.
